# Two Novel Variants in the *CHRNA2* and *SCN2A* Genes in Italian Patients with Febrile Seizures

**DOI:** 10.3390/genes15111407

**Published:** 2024-10-30

**Authors:** Radha Procopio, Monica Gagliardi, Mariagrazia Talarico, Francesco Fortunato, Ilaria Sammarra, Anna Caterina Procopio, Paola Roncada, Donatella Malanga, Grazia Annesi, Antonio Gambardella

**Affiliations:** 1 Department of Medical and Surgical Sciences, Neuroscience Research Center, Magna Graecia University, 88100 Catanzaro, Italy; radha.procopio@unicz.it; 2 Institute of Neurology, Department of Medical and Surgical Sciences, Magna Graecia University, 88100 Catanzaro, Italy; mary.talarico21@gmail.com (M.T.); francescofortunato@unicz.it (F.F.); ilaria.sammarra@unicz.it (I.S.); a.gambardella@unicz.it (A.G.); 3 Department of Health Sciences, Magna Graecia University, 88100 Catanzaro, Italy; procopioannacaterina@unicz.it (A.C.P.); roncada@unicz.it (P.R.); 4 Laboratory of Molecular Oncology, Department of Experimental and Clinical Medicine, Magna Graecia University, 88100 Catanzaro, Italy; malanga@unicz.it; 5 Interdepartmental Center of Services (CIS), Magna Graecia University, 88100 Catanzaro, Italy; 6 Institute for Biomedical Research and Innovation, National Research Council, 87050 Mangone, Italy; grazia.annesi@irib.cnr.it

**Keywords:** *CHRNA2*, nicotinic acetylcholine receptor (nAChR), *SCN2A*, voltage-gated sodium channel (Nav) 1.2, febrile seizures

## Abstract

Background: Febrile seizures (FSs) are the most common form of epilepsy in children aged between six months and five years. The exact cause is unknown, but several studies have demonstrated the importance of genetic predisposition, with increasing involvement of receptors and ion channels. The present study aims to identify novel pathogenic variants in Italian patients with FSs. Methods: We performed targeted panel sequencing in a cohort of 21 patients with FSs. In silico analysis was performed to predict the pathogenic role of the resulting variants. Results: We found two novel variants segregating in two families with FSs: c.1021C>G (p.Leu341Val) in the *CHRNA2* gene and c.140A>G (p.Glu47Gly) in *SCN2A*. Conclusions: The c.1021C>G (p.Leu341Val) variant leads to a codon change of highly conserved leucine to valine at position 341 and is located in segments M3 of the subunit, which is important for channel gating. The c.140A>G (p.Glu47Gly) variant causes a substitution of glutamic acid with glycine at position 47 of the protein, which is highly conserved across the species. Moreover, it is located in the N-terminal domain, a region commonly affected in ASD, which impacts the inactivation kinetics and voltage dependence of steady-state activation. Further analyses are needed to better explain the role of *CHRNA2* and *SCN2A* in the development of febrile seizures.

## 1. Introduction

Febrile seizures (FSs) are the most common childhood convulsive event, with a prevalence of 2–5% in Western countries [1] and 7–8% in Asian populations [2]. FSs are a neurological condition characterized by fever without a recognized cause, like infection, trauma, metabolic disease, or intoxication, that affect children aged six months to five years, with a peak incidence at 18 months [3]. FSs are categorized into simple FSs, complex FSs, and febrile status epilepticus, based on the type, length, and frequency of convulsions: (1) a simple FS is a generalized seizure that typically lasts for <15 min, does not recur within 24 h, and is caused by a particular illness (like respiratory or digestive infection); (2) a complex FS is a focal seizure, lasting between 15 and 30 min, that recurs within 24 h, and (3) febrile status epilepticus is a seizure that lasts more than 30 min. The majority of FSs are simple (70–80% of cases), 25% are complex, and 5% are febrile status epilepticus [4]. Other signs of an FS include loss of consciousness, eyes rolling back, cyanosis, irregular breathing, and jerking of the extremities [5].

The cause seems to be multifactorial, however increasing evidence supports the crucial role of genetic factors in the pathogenesis of FSs, with approximately one-third of children with FSs having a positive familial history [6]. Several loci have been verified, including FEB1, FEB2, FEB3, FEB5, FEB7, FEB8, FEB9, and FEB10, but only few genes have been identified on them, like *SCN1A* [7], *ADGRV1* [8], and *GABRG2* [9]. Therefore, the association between FSs and both receptors and ion channels now seem clear.

Acetylcholine (ACh) is a neuromodulator, released by neuronal cells called cholinergic cells, that works as an excitatory neurotransmitter in the brain [10]. When ACh binds to specific sites called nicotinic acetylcholine receptors (nAChRs), it switches their functional state, allowing the passage of sodium ions from the outside to the inside of the cell.

The nAChRs are pentameric ligand-gated channels, composed of various combinations of α and β subunits with different locations, pharmacological properties, and physiological roles. Each subunit consists of a sizable extracellular N-terminal domain (ECD), three hydrophobic transmembrane α-helix segments (M1-M3), a long intracellular domain (ICD), a fourth transmembrane region (M4), and a short variable extracellular C-terminal segment [11,12].

The large ECD contains the binding sites for ACh. The M1-M4 segments are connected by small loops and assembled in a massive transmembrane domain (TMD); the M2 hydrophobic segments form the ionic pore, with some contribution from M1, determining the ion selectivity of the receptor and the energy level of allosteric transition. The ICDs differ among subunits, regulating assembly, function, trafficking, and interactions with intracellular proteins through post-translational modifications [11,12]. Broadly, nAChRs are classified into muscle- and neuronal-type. Neuronal nAChRs are distributed in the central and peripheral nervous systems, as well as in several types of non-neuronal cells. nAChRs play crucial roles in several physiological processes, including cognition, autonomic functions, drug addiction, and inflammation [13]. Dysregulation of nAChR activity is associated with a spectrum of human disorders, including Parkinson’s disease, Alzheimer’s disease, inflammatory disorders, substance dependence, and epilepsy [14].

The principal subunits found in the brain are α2, α4, and β2, encoded by *CHRNA2*, *CHRNA4*, and *CHRNB2* genes, respectively; in detail, α2-nAChR is primarily expressed in the central nervous system and seems to be located on the postsynaptic cell membrane [15].

Mutations in these genes are typically associated with sleep-related hypermotor epilepsy (SHE), previously known as autosomal dominant nocturnal frontal lobe epilepsy (ADNFLE). In particular, several mutations were found in *CHRNA4* [16] and *CHRNB2* [17], whereas only four variants were reported in *CHRNA2* [18,19,20], including one in a family with self-limited familial infantile epilepsy (SeLFIE), previously known as benign familial infantile seizures (BFISs) [21]. Functional studies indicate that hyperstimulation of nAChRs is the main pathogenic mechanism causing seizures [22].

Instead, the voltage-gated sodium channel (Nav) family consists of nine isoforms (Nav1.1–Nav1.9), composed of a main α-subunit and one or more accessories of β-subunits. The large α-subunit, which is highly conserved, is sufficient for channel functionality and is organized in four domains (I-IV), each containing six transmembrane segments (S1–S6). The voltage sensor is located at the level of the S4 segment, while the ionic pore with the selectivity filter is formed by the loop between the S5 and S6 helices [23]. Accessory β-subunits are a single transmembrane region that contributes to adhesion through interaction with cell adhesion or intracellular matrix proteins [24].

Nav channels play a crucial role in the initiation, propagation, and regulation of action potentials in neuronal networks. Altered excitability leads to the dysfunction of several brain regions, causing genetic epilepsy in most cases [23].

The nine Nav channels are encoded by *SCN1A*-*SCN5A* and *SCN8A*-*SCN11A* genes and are expressed in several mammalian tissues. Nav1.1, Nav1.2, Nav1.3, and Nav1.6 are highly expressed in the central nervous system, while Nav1.7, Nav1.8, and Nav1.9 are mostly restricted to the peripheral nervous system. Nav1.4 is expressed in adult skeletal muscles and Nav1.5 in the myocardium [25].

In detail, Nav1.2, encoded by *SCN2A*, is widely expressed in all developmental ages, including prenatally. The steps between the different conformational states (closed, open, inactivated) of the channel are involved in the generation and propagation of the action potentials in excitatory neurons [26].

Pathogenic variants in *SCN2A* are one of the most common causes of neurodevelopmental disorders. Since the first description in patients with self-limited familial neonatal-infantile epilepsy (SeLFNIE) [27,28], previously known as benign familial neonatal-infantile seizures (BFNISs), the phenotypic spectrum has expanded considerably, including developmental and epileptic encephalopathies (DEEs), autism spectrum disorder (ASD), and intellectual disability (ID) with or without seizures [29].

In patients with epilepsy, the SCN2A variants lead to increased sodium channel activity and are typically missense heterozygous variants, with the majority (82%) inherited for SeLFNIE and de novo for DEE. Even in ASD/ID phenotypes, variants are de novo, however there is a higher prevalence of truncating mutations resulting in nonfunctional Nav1.2 channels.

To date, more than 300 patients and about 100 epileptogenic mutations have been reported, recognized mostly at the level of the selectivity filter, the pore, and the voltage sensor [30]. Moreover, variants in *SCN2A* have been reported in monozygotic twin with genetic epilepsy with febrile seizures plus (GEFSs+) [31] and in a patient with FSs and afebrile seizures [27].

Here, we performed targeted panel sequencing in an Italian cohort of 21 patients with FSs. Two novel missense variants, one in *CHRNA2* and one in *SCN2A*, were found in two unrelated families.

## 2. Materials and Methods

A meticulous review of 21 patients’ medical records and direct interviews were conducted. All cases originating from south of Italy (Calabria and Sicily) were classified as simple FSs, in accordance with the latest International League Against Epilepsy (ILAE) recommendations, because they experienced fever in childhood accompanied by generalized seizures that were not caused by central nervous system infection and did not meet the criteria for other acute symptomatic seizures.

Among the affected children (13 males and 8 females), 15 had a positive family history. The first seizure occurred between 3 and 48 months (mean 20.25 ± 12.27). Eight patients had only one seizure, four patients had two seizures, two patients had three seizures, and seven patients had four or more seizures.

The study was conducted in accordance with the declaration of Helsinki and approved by the ethics committee of University Magna Graecia of Catanzaro.

Written informed consent was obtained from each participant. Genomic DNA was extracted from peripheral blood using a Wizard Genomic DNA extraction kit, following the manufacturer’s instructions (Promega, Madison, WI, USA).

Starting from 10 ng of gDNA, library preparation was performed through the Ion Ampliseq library Kit Plus (Life Technologies, Carlsbad, CA, USA) with an AmpliSeq custom panel of 39 genes (previously associated with the development of epilepsy and listed in Table S1).

Ion PGM Template OT2 200 kit and Ion OneTouch-2 Instrument were used for template preparation.

The libraries were sequenced on Ion PGM-Dx sequencer (Thermo Fisher, Vacaville, CA, USA).

Torrent Suite Software v5.12 was used to perform data analysis and filtering the variants according to coverage ≥ 100, quality score ≥ 30, and frequency ≥ 5%, followed by the dbSNP141, 1000 Genomes Project datasets, and the 5000Exome database. Moreover, the common polymorphic variants observed in the general population (MAF > 0.5%) were removed. Finally, the resulting variants were filtered after functional annotation SIFT (http://sift.jcvi.org, accessed on 15 August 2024) and/or PolyPhen-2 (http://genetics.bwh.harvard.edu/pph2/, accessed on 15 August 2024) algorithms.

The variants obtained were validated using a BigDye Terminator chemistry ver. 3.1 (Life Technologies) on an ABI 3500 Genetic Analyzer (Life Technologies). Segregation analysis was performed in the parents of the probands.

Mutation Taster (https://www.mutationtaster.org/, accessed on 15 August 2024) and Polyphen-2 were examined to predict the potential pathogenic impact of the identified variant on protein function. The REVEL Score (a score > 0.5 is considered pathogenic), CADD-phred Score (a score > 20 is considered pathogenic), and MetaDome Score (https://stuart.radboudumc.nl/metadome/, accessed on 15 August 2024 a score between 0 and 0.5 is considered intolerant, between 0.5 and 0.7 is considered slightly intolerant, and >0.7 is considered neutral) were evaluated to ascertain the probability of missense variant pathogenicity.

The American College of Medical Genetics and Genomics (ACMG) guidelines [32] were applied to classify the variant.

Finally, the three-dimensional structure of the neuronal acetylcholine receptor was implemented using the prediction software SWISS-MODEL Repository of 3D Protein Structures and Models [33,34,35,36,37]. The wildtype and mutant protein structure were aligned using PyMOL software (version 2.5.7), selecting the human acetylcholine receptor model [38].

## 3. Results

### 3.1. Clinical Characteristic

#### 3.1.1. Family 1

**Figure 1:**
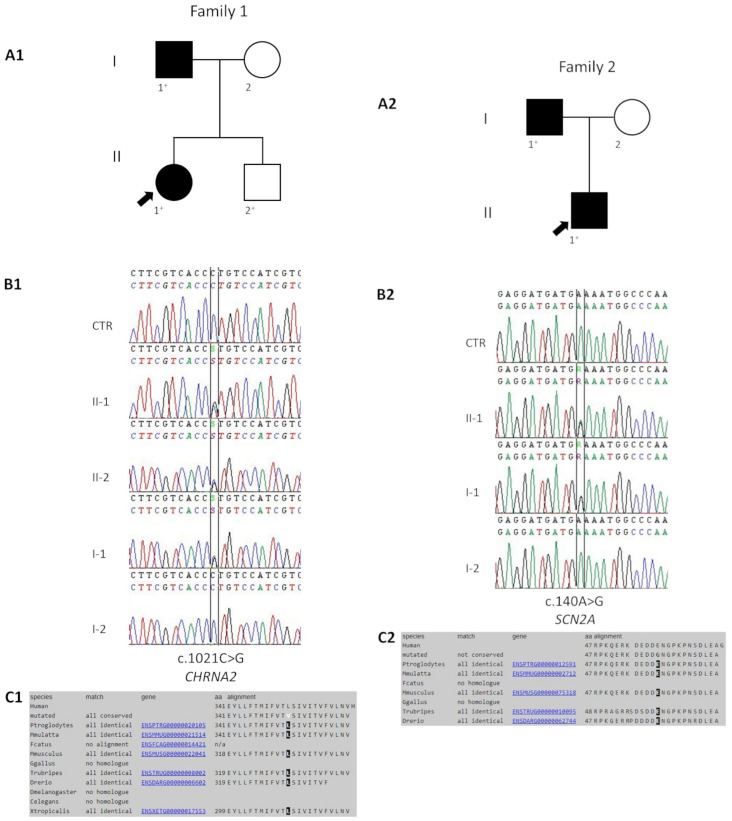
Pedigree of the families recruited, CHRNA2 and SCN2A sequence variants and conservation. (**A1**) Pedigree of Family 1. (**A2**) Pedigree of Family 2. The fully filled symbols represent the affected individuals; unfilled symbols, unaffected; arrow, proband; plus, individuals with variants. (**B1**) Electropherogram shows the wildtype sequence (at the top), the c.1021C>G variant in *CHRNA2* in proband (II-1), her unaffected brother (II-2) and affected father (I-1) (middle), and the wildtype sequence in her unaffected mother (I-2) (at the bottom). (**B2**) The wildtype sequence (at the top), the c.140A>G variant in *SCN2A* in heterozygous proband (II-1) and his affected father (I-1) (middle), and the wildtype sequence in his unaffected mother I-2 (at the bottom). (**C1**) Alignment of CHRNA2 proteins shows high evolutionary conservation of Leucine 341. (**C2**) Alignment of SCN2A proteins shows high evolutionary conservation of glutamic acid 47.

The proband (Figure 1(A1) (II-1)) was a 36-year-old female born at term after a normal pregnancy and delivery. At the age of 14 months, she experienced her first generalized tonic–clonic seizure that lasted 4 min, with a fever of 40 °C. Upon arrival at the emergency department, the patient had fully recovered. One month later, she had a second and last seizure with the same characteristics. Physical and neurological evaluations were normal. In magnetic resonance images (MRIs) of the brain and on interictal electroencephalogram (EEG), no evident abnormalities were observed. She was not treated. Her growth and developmental milestones were appropriate for her age. She had no other health problems.

The proband’s 59-year-old father (Figure 1(A1) (I-1)) had three generalized tonic–clonic seizures; the first at the age of 10 months, the second at the age of 11 months, and the last one at the age of 16 months, all provoked by fever. The EEG and MRIs performed during infancy showed no abnormalities. He did not experienced seizures later in life. His development was completely normal and he completed his college education successfully.

The proband’s brother (Figure 1(A1) (II-2)) was a 35-year-old healthy male, born at term after an uneventful pregnancy and delivery. He only experienced untreated migraine without aura, starting from the age of 10. His psychomotor development was normal and he attended school with good results.

The proband’s 58-year-old mother (Figure 1(A1) (I-2)) reported to have been affected by hot water epilepsy (HWE) in her adolescence, characterized by tremors, cyanosis of the extremities, tachycardia, and breathing difficulties triggered by immersion of the body parts in hot water. Since the age of 22, all seizure activity has been absent. Physical examination, repeated EEGs, and brain MRIs were normal. Her psychomotor development was normal.

#### 3.1.2. Family 2

The proband (Figure 1(A2) (II-1)) was a 19-year-old male born at term after an uneventful pregnancy and delivery. He had his first generalized tonic–clonic febrile seizure at the age of 12 months and 13 days, and his last one at the age of 2 years. In total, he experienced four seizures, which were all provoked by fever. No anti-seizure medications were administered. His physical and neurological examinations were normal. The EEG and MRI showed no abnormalities. His growth and developmental milestones were normal; head control was achieved at 3 months, sitting alone at 7 months, walking at 14 months, and speaking at 13 months. He had no other health or developmental problems.

The proband’s father (Figure 1(A2) (I-1)) was a 47-year-old man. He was born at term after a normal pregnancy and delivery. At the age of 13 months, he experienced a generalized tonic–clonic febrile seizure. In total, he experienced three febrile seizures, one per year. Brain MRI and EEG performed during infancy was reported to be normal. He did not experienced seizures later in life. His psychomotor and cognitive development was completely normal, with good school performance.

The proband’s mother (Figure 1(A2) (I-2)) was a healthy woman aged 45 years, with no seizures or other neurological diseases. Neurological examination was normal and no other abnormalities were found on physical examination.

### 3.2. Genetic Data

The Ion Torrent platform was used to perform next generation sequencing. After filtering of the variants as described in patients and methods section, we excluded all synonymous, 5′UTR, and 3′UTR variants. The analysis revealed two novel missense variants in two unrelated cases: c.1021C>G (p.Leu341Val) in the *CHRNA2* gene and c.140A>G (p.Glu47Gly) in *SCN2A*.

**Figure 2:**
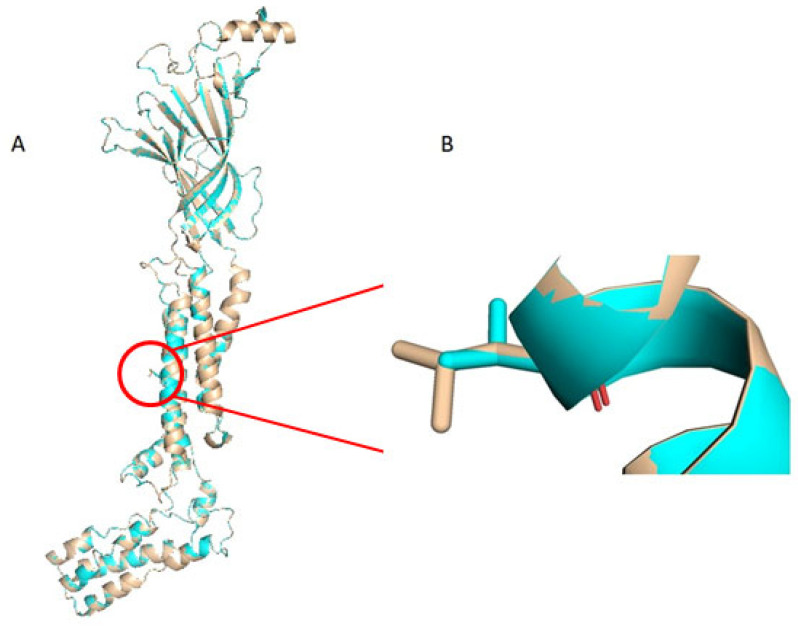
Three-dimensional model of CHRNA2 protein. (**A**) Superposition between the wildtype and mutant protein model. (**B**) p.Leu341Val variant overlay, with leucine colored light brown and valine colored light blue.

The c.1021C>G (p.Leu341Val) was found in the *CHRNA2* gene in a heterozygous state of the II-1 (Figure 1(B1)). This variant leads to a codon change of leucine to valine at position 341, which is highly conserved across the species (Figure 1(C1)). The variant c.1021C>G (rs750301958) was not reported in previous studies and the database of gnomAD revealed a rare minor allele frequency (MAF) of 0.00001177. Segregation analysis in the rest of the family revealed the presence of the same variant in her affected father and unaffected brother, but not in her mother (Figure 1(B1)). Mutation Taster, PolyPhen2, and SIFT predicted a damaging role of the c.1021C>G variant. Moreover, REVEL and CADD-phred established the probable pathogenicity of the variant with scores of 0.696 and 25.80, respectively. The p.Leu341Val is located in the hydrophobic transmembrane α-helix segments M3, in a position that is “slightly tolerant” according to MetaDome, with a score of 0.57. The computational modelling study, performed to assess the structural impact of the p.Leu341Val variant on the three-dimensional structure of the CHRNA2 protein, revealed a decreased value of thermodynamic stability in the mutant protein, with a ΔΔG of −0.678 kcal/mol (Figure 2) compared to the wildtype form [39,40]. We classified the variant as “likely pathogenic” according to the ACMG guidelines (PM1+PM2+PP1+PP3).

The c.140A>G (p.Glu47Gly) was found in exon 2 of *SCN2A* (NM_001040143.1) in a heterozygous state of the II-1 (Figure 1(B2)). Sanger sequencing analysis confirmed the presence of this variant in his affected father, while it was absent in the unaffected mother (Figure 1(B2)). The c.140A>G variant has a very low minor allele frequency (MAF) of 6.195 × 10^−7^ in the gnomAD database, and it has never been reported in previous studies. This variant leads to a substitution of glutamic acid with glycine in position 47 of the protein, which is strongly conserved in several eukaryotic sodium channels (Figure 1(C2)). According to Mutation Taster and PolyPhen2, the c.140A>G variant is disease causing. REVEL and CADD-phred predicted a pathogenic role of the variant with scores of 0.65 and 26.40, respectively. It is located in N-terminal unstructured domain and is in a position that is “slightly tolerant” according to the MetaDome score. Following the ACMG guidelines, we classified the variant as “likely pathogenic” (PM1+PM2+PP1+PP3).

No other pathogenic variants were detected.

## 4. Discussion

In the present study, we reported two novel missense variants, c.1021C>G (p.Leu341Val) in *CHRNA2* and c.140A>G (p.Glu47Gly) in *SCN2A,* in two families with FSs.

Specifically, c.1021C>G (p.Leu341Val) was found in *CHRNA2* in a girl and her father affected by FSs. Her brother also carried the variant, but he had never suffered from any type of epilepsy; therefore, the p.Leu341Val variant was inherited with incomplete penetrance, similarly to some previously reported cases [41,42]. Instead, the proband’s mother, affected by HWE in her adolescence, did not harbor the variant, thus further analysis is needed to better understand the pathophysiology of her condition.

*CHRNA2* is located on chromosome 8p21.2, contains seven exons, and encodes the α2 subunit of nAChRs, which regulates neuronal excitability after the binding of Ach.

Typically, mutations in *CHRNA2*, together with *CHRNA4* and *CHRNB2*, are associated with the development of the autosomal dominant SHE. However, a mutation in *CHRNA2* was also found in a family with SeLFIE [21]. No variants of FSs are reported in the Human Gene Mutation Database (https://www.hgmd.cf.ac.uk/ac/all.php, accessed on 15 August 2024). Functional studies have demonstrated that mutations in nAChRs tend to have a gain-of-function (GoF) effects, increasing the receptor’s sensitivity to Ach which leads to seizures [14].

Our c.1021C>G (p.Leu341Val) variant, reported in the gnomAD database to have a very low MAF (0.00001177), is predicted to be a pathogenic variant according to in silico analysis. It is located in the hydrophobic transmembrane α-helix segment M3 of the CHRNA2, where it causes slight thermodynamic destabilization according to our computational analyses.

Advanced microscopy techniques have revealed that all nAChRs share the same topology, characterized by four α-helix transmembrane regions (M1–M4) and extracellular N- and C-terminal domains. The five M2s of each nAChR assemble the inner wall of the ionic pore, surrounded by the M1 and M3 helices and further externally, the M4 [43].

Most of the mutations found so far in the genes encoded for nAChRs are located in the M2 domain, thus are considered crucial for the development of SHE. However, recent studies have shown that three variants found in the M3 domain of *CHRNB2*, namely p.Leu301Val, p.Val308Ala, and p.Ile312Met, play an important role in channel gating, increasing the sensitivity to Ach [44,45] and resulting in the same GoF effect proposed in all previously described SHE mutations. Considering this evidence, we can speculate that our CHRNA2 variant p.Leu341Val, located in the crucial M3 domain, is the cause of FSs development in the family described here.

The reason why the p.Leu341Val variant causes FSs rather than SHE remains to be clarified. It has been observed that the clinical spectrum associated with p.Leu301Val and p.Val308Ala appears to be mostly the benign type of SHE, with no other neurological and psychiatric features [45], whereas p.Ile312Met causes a more severe form of SHE, with a decrease in cognitive function [44,46]. This could be explained by the fact that the residual Ile312 is inserted at the beginning of the α-helix of M3 and forms a bifurcated hydrogen bond with the CO groups of Val308 and Thr309, conferring stability to the domain [47,48]. Moreover, it has been demonstrated that the interactions between TM3 and TM2 are strengthened by longer amino acid side chains to reduce channel opening, whereas shorter amino acid side chains facilitate gate opening and increase Ach sensitivity [49]. Therefore, as it is in a position that is not crucial for the stability of the α-helix of M3, as confirmed by our thermodynamic stability analyses, and as leucine and valine have very similar amino acid side chains, we can presume that our p.Leu341Val variant leads to a milder phenotype, such as FSs, although further functional analysis are needed to confirm our hypothesis.

Regarding the other variant, c.140A>G (p.Glu47Gly) was found in the *SCN2A* gene in a young male and his father, both affected by FSs. The variant was located in the N-terminal domain of the Nav1.2 channel, and is considered to be a pathogenic variant according to all of the prediction tools consulted.

*SCN2A* is located on chromosome 2q24.3. It contains 26 exons and encodes the α subunit of the voltage-gated sodium channel Nav1.2. Pathogenic variants in *SCN2A* are associated with a spectrum of epileptic and neurodevelopmental disorders, i.e., SeLFNIE, ID, and ASD, presenting an autosomal dominant inheritance [50].

Variants often occur in functionally crucial and highly conserved regions of the protein. The amino acid substitutions associated with DEE, ASD, and ID are typically between physicochemically dissimilar amino acids, causing severe disease, in contrast to the amino acid substitutions observed in SeLFNIE that are between similar amino acids, which cause less severe disease [51].

Existing functional analyses have demonstrated that SCN2A truncating/deletion variants, producing nonfunctional Nav1.2 channels, have a loss-of-function (LoF) effects and are associated with later-onset seizures, ASD, and ID, whereas SCN2A missense variants with gain-of-function (GoF) effects are related to early self-limited familial epilepsy [26,52]. This GoF missense improves neuronal excitability, leading to a different seizure phenotype. However, the expression of Nav1.2 in excitatory neuron axons, which allows AP initiation and propagation, decreases with time. Nav1.2 is largely replaced by Nav1.6 (*SCN8A*) at approximately 1–2 years of age, while Nav1.2 is thought to contribute to backpropagation of Aps to somatodendritic compartments [53]. Therefore, the increased excitability during the early stage ends once the brain matures, consistent with the positive prognosis of SeLFNIE patients.

Our novel SCN2A c.140 A>G (p.Glu47Gly) variant presents unique peculiarities.

Firstly, it was identified in a family affected by febrile seizures. To our knowledge, no other SCN2A variants were reported in FSs cases; only two variants were found in two clinical subsets of FSs, i.e., GEFS+ and FSs with afebrile seizures [27,31]. Although further functional studies are required to confirm its pathogenic role, the identification of this variant in a family with FSs broadens the phenotypic spectrum of the *SCN2A* gene. Moreover, a recent genome-wide association study of FSs conducted on 7635 cases and 83,966 controls confirmed four susceptibility loci for FSs, including the sodium channel gene SCN2A [54].

Finally, c.140A>G (p.Glu47Gly) is an inherited missense variant, like all variant causing early self-limited familial epilepsy, so we could hypothesize that it has a GoF effect, as reported in functional studies of the literature. However, it affects a highly conserved amino acid in the N-terminal region, a typical domain hit in ASD. Indeed, the only missense variants found in the N-terminal domain are p.Asp12Asn and p.Asp82Gly, which cause a loss of Nav1.2 function, altering the inactivation kinetics and voltage dependence of steady-state activation, respectively [55].

Consequently, our novel c.140A>G (p.Glu47Gly) variant is the first example of an N-terminal pathological variant found in self-limited epilepsy.

Certainly, our study has some limitations; the small number of FSs families included, the small number of genes in our targeted panel, and the absence of functional studies. However, the data illustrated here, which represent the first association between *CHRNA2* and FSs phenotype and support the role of *SCN2A* in the development of FSs, suggest consideration of the role of these genes in the development of febrile seizures, and a testing theme for all epileptic phenotypes.

## Data Availability

The data that support the findings of this study are available from the corresponding author (M.G.) upon reasonable request.

## Supplementary Materials

The following supporting information can be downloaded at: https://www.mdpi.com/article/10.3390/genes15111407/s1, Table S1: Targeted panel sequencing.
